# A Reduced GO-Graphene Hybrid Gas Sensor for Ultra-Low Concentration Ammonia Detection

**DOI:** 10.3390/s18093147

**Published:** 2018-09-18

**Authors:** Chang Wang, Shaochong Lei, Xin Li, Shixi Guo, Ping Cui, Xianqi Wei, Weihua Liu, Hongzhong Liu

**Affiliations:** 1 Department of Microelectronics, School of Electronics and Information Engineering, Xi’an Jiaotong University, Xi’an 710049, China; wangc254@163.com (C.W.); leisc@mail.xjtu.edu.cn (S.L.); guoshixi@stu.xjtu.edu.cn (S.G.); cuiping_dc@163.com (P.C.); wei.wxq@163.com (X.W.); lwhua@mail.xjtu.edu.cn (W.L.); 2 Guangdong Shunde Xi’an Jiaotong University Academy, Foshan 528300, China; 3 Research Institute of Xi’an Jiaotong University, Hangzhou 311215, China; 4 State Key Laboratory for Manufacturing Systems Engineering, Xi’an Jiaotong University, Xi’an 710049, China; hzliu@mail.xjtu.edu.cn

**Keywords:** graphene, reduced graphene oxide (RGO), sensitivity, signal-to-noise (SNR), gas sensor, detection limit

## Abstract

A hybrid structure gas sensor of reduced graphene oxide (RGO) decorated graphene (RGO-Gr) is designed for ultra-low concentration ammonia detection. The resistance value of the RGO-Gr hybrid is the indicator of the ammonia concentration and controlled by effective charge transport from RGO to graphene after ammonia molecule adsorption. In this hybrid material, RGO is the adsorbing layer to catch ammonia molecules and graphene is the conductive layer to effectively enhance charge/electron transport. Compared to a RGO gas sensor, the signal-to-noise ratio (SNR) of the RGO-Gr is increased from 22 to 1008. Meanwhile, the response of the RGO-Gr gas sensor is better than that of either a pristine graphene or RGO gas sensor. It is found that the RGO reduction time is related to the content of functional groups that directly reflect on the gas sensing properties of the sensor. The RGO-Gr gas sensor with 10 min reduction time has the best gas sensing properties in this type of sensor. The highest sensitivity is 2.88% towards 0.5 ppm, and the ammonia gas detection limit is calculated to be 36 ppb.

## 1. Introduction

Ammonia gas is widely present in the surrounding environment. It is a colorless, irritating odor and highly toxic gas, therefore, the detection of ammonia is very important due to the potential threat to people’s health posed by ammonia [1,2]. Meanwhile, since ammonia is a byproduct of protein metabolism, the detection of ammonia can help clinicians assess diseases and state of health [3,4]. Due to the merits of non-invasion, quickness, convenience, and repeatability, exhaled ammonia detection has attracted more attention for early diagnosis of many diseases such as hepatic injury, kidney diseases, *Helicobacter pylori* infection and halitosis [5,6,7,8,9]. As reported, the concentration of ammonia in human exhaled breath is approximately in the range of 50 parts per billion (ppb) to 5 parts per million (ppm) [4]. In order to improve previous medical testing methods, there is an urgency to design an ultra-low concentration ammonia sensor. According to the operating mechanism of gas sensors, there are mainly two kinds of gas detection technology, namely, spectrum analysis and gas sensor systems [10,11,12,13]. The gas sensor system technology is suitable for online and timely detection, while, spectrum analysis methods such as spectrometry and chromatography have a long and complicated analysis cycle [10].

Different materials are under study by different research groups for gas sensor applications. The most widely sensitive materials for gas sensors are the metal oxide materials, including ZnO, SnO_2_, NiO, and so on [14,15,16]. These metal oxide semiconductors usually have very excellent gas sensing properties. Additionally, metal hydroxides [17], metal sulfides [14], binary metal oxides [18], and other materials have been extensively and studied as-sensitive materials, and usually have better gas sensing properties.

Graphene and its derivatives are considered as promising gas sensor materials in the field of ultra-high sensitivity detection because of their high surface area and high carrier mobility [19,20,21,22,23]. Research aimed at improving the gas sensing properties of graphene to detect the ultra-low concentration ammonia gas is an emerging field. Graphene is functionalized with metal oxides (such as SnO_2_, ZnO, Cu_2_O, WO_3_) to form hybrid nanostructures sensors [24,25,26,27,28,29], because the high surface area of graphene may cause synergetic effects in achieving a good gas response at room temperature while blended with metal oxides, especially due to formation of a p-n junctions, and the resulting novel sensors may exhibit better performance than those of the individual materials [29]. Graphene has functionalized by decoration with metal nanoparticles (NPs) (including AuNPs, AgNPs) for gas sensing [30,31,32,33,34,35]. The sensitivity depends on the degree of coverage of the metal NPs on the graphene [35]. Su et al. have prepared an ammonia sensor with Pd/SnO_2_/RGO ternary composite, the ammonia gas sensitivity of which reached 25 ppm [36]. An ammonia gas sensor prepared by RGO and P3TH, also could detect 10 ppm concentrations of ammonia [37]. Both of them have used as gas sensing material modified RGO as the adsorption layer with RGO as the conductive sensing material whose conductivity is far less than that of graphene. However, the sensitivity of the above sensors does not reach ultra-low detection concentrations.

The electron transport of graphene is highly sensitive to most gas molecules owing to its high mobility and presence of a large number of surface atoms in its 2D structure [38,39,40]. The devices based on graphene are expected to have relatively low Johnson noise and 1/f noise because of their high conductance and low crystal defect density, respectively [39,41]. However, graphene is intrinsically rather inert owing to its few gas molecule adsorption sites [42]. On the other hand, RGO has inherent chemical defects, and attached functional groups which can provide a large number of adsorption sites beneficial to adsorbing ammonia gas, but the conductivity is much worse compared to that of graphene, meanwhile, Johnson noise and 1/f noise of RGO are higher than that of pristine graphene.

This paper proposes a new RGO-Gr hybrid structure based on pristine CVD graphene decorated with RGO to combine the advantages of both graphene and RGO, in order to obtain good gas sensitivity and low noise characteristics. The effects of different RGO reduction time on gas sensing properties have also been studied.

## 2. Materials and Methods

### 2.1. Preparation of Graphene and RGO

**Figure 1:**
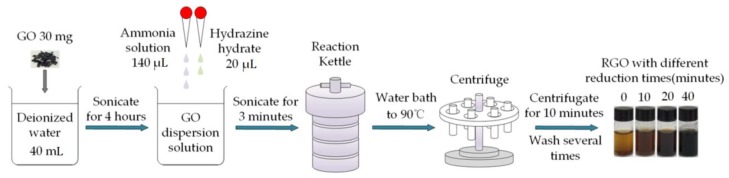
Synthesis flowchart of RGO with four different reduction times.

Graphene was synthesized on copper foil (#13382, 99.8%, Alfa Aesar, Haverhill, MA, USA) by a chemical vapor deposition (CVD) method [43]. RGO was prepared by a multiple steps process. Initially, GO (30 mg, #XF002-1, XF Nano Inc., Nanjing, China) was dispersed in deionized water (40 mL) and sonicated for 4 h. Next, after adjusting the pH value of the solution by adding 25% ammonia solution (140 μL), 80% hydrazine hydrate (20 μL) was added as a reducing agent to the solution which was sonicated for 3 min. Then, the solution was transferred into a reaction kettle and heated to 90 °C using the water bath for different reduction times to get different samples. Finally, the obtained solution was centrifuged and washed with deionized water several times and the final product was obtained. The final samples were labeled as RGO0 (GO), RGO10, RGO20 and RGO40, corresponding to the samples prepared using different reduction times (0, 10, 20 and 40 min). For more clarification, the synthesis process of RGO is schematically described in Figure 1.

### 2.2. Fabrication of RGO-Gr Hybrids Gas Sensor

**Figure 2:**
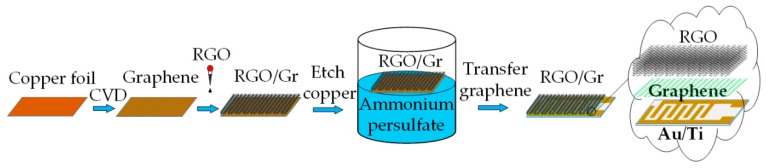
Fabrication process of the RGO-Gr hybrid gas sensors.

The fabrication process of the RGO-Gr hybrid gas sensors is shown in Figure 2. Firstly, the RGO solution was evenly spin coated on the surface of CVD graphene on copper foil, which was placed on a heating table and dried for 5 min at 50 °C. Secondly, the copper foil was etched away with prepared ammonium persulfate. Thirdly, the RGO-Gr hybrid film was obtained and washed several times in deionized water. Finally, RGO-Gr hybrid was transferred to a fresh interdigital electrode (IDE) and the RGO-Gr hybrid gas sensor device was thus obtained. The graphene on the IDEs was used as the conductive layer while RGO decorated on graphene was used as the ammonia molecule adsorption layer.

In this graphene transfer process, RGO replaced PMMA (polymethyl methacrylate) as the protective layer of graphene, which not only avoided the organic contamination caused by the introduction of PMMA, but also reduced the damage to the graphene by reducing the experimental steps. Six sensors have been made to conduct comparative research. The sensing materials of the first two sensors were pristine CVD graphene and pristine RGO (RGO0), namely Sensor-1 and Sensor-2, respectively. The sensing materials of the latter four sensors were all RGO-Gr hybrids structures with four different reduction times of RGO. The six sensors could be classified into three kinds as listed in Table 1.

### 2.3. Material Characterization

The morphology and structure of the graphene were characterized with a scanning electron microscope (SEM, S-4800, Hitachi, Tokyo, Japan). The graphene transferred to the IDEs was measured at room temperature with a Raman spectrometer (JY HR800, Horiba, Lille, France) at 514 nm with an output power of 20 mW. X-ray photoelectron spectroscopy (XPS) (Axis Ultra DLD, Kratos Inc., Manchester, UK) was used to determine the elemental composition and state of the composites.

### 2.4. Gas Sensing Detection

**Figure 3:**
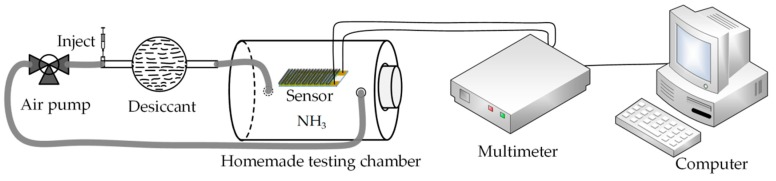
Schematic diagram of the gas sensing system.

Ammonia gas detection experiments were carried out in a simple homemade testing chamber, while the total volume of chamber, drying tube and filling pipe was measured and calculated as 4.67 L as shown in Figure 3. The drying pipe filled with potassium hydroxide as a desiccant was used to convert injected ammonia solution into dry ammonia gas which was passed into the chamber by an air pump (MEDOVP0125-V1005-P2-1411, Nitto Kohki, Tokyo, Japan). Details of the calibration and control of ammonia concentration and humidity are provided in the electronic Supporting Information (SI). The sensor temperature and the relative humidity (RH) in the test chamber were room temperature (about 25 °C ± 2 °C) and 30 ± 4%, respectively. The resistances of proposed gas sensors were detected and recorded by a multimeter (2000, Keithley Instruments, Cleveland, OH, USA) and a computer running the Lab-VIEW software for automatic data storage under different ammonia concentration conditions. The concentrations of ammonia gas were varied from 0.5 to 50 ppm.

## 3. Results and Discussion

### 3.1. Characterization of the Morphology and Structure of Materials

**Figure 4:**
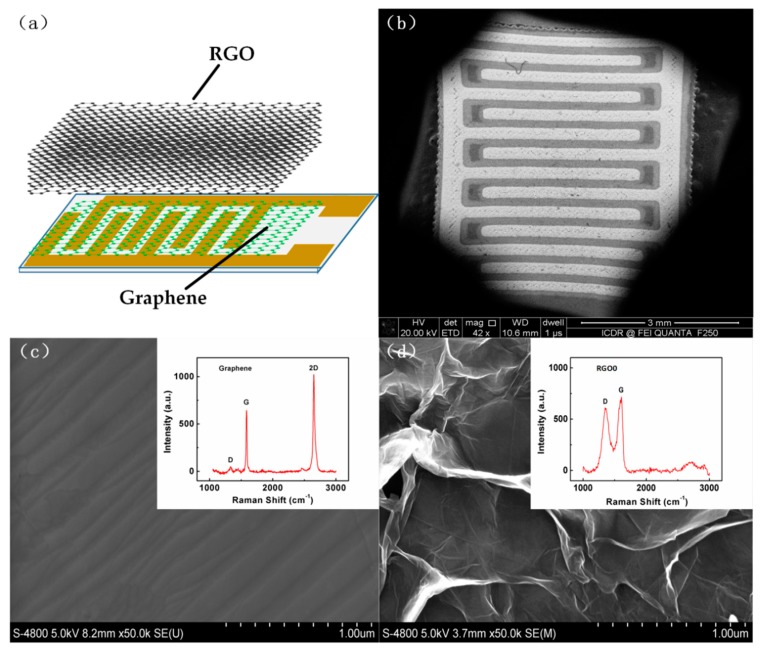
(**a**) Schematic diagram of RGO-Gr hybrids gas sensors; (**b**) SEM image of CVD Graphene on IDE electrode; SEM image and Raman spectra of the (**c**) graphene and (**d**) RGO0.

Figure 4a illustrates schematic diagram of the RGO-Gr hybrid sensor. Graphene on the IDEs is used as the conductive layer, and RGO decorated on the graphene is used as gas adsorption layer, which forms the hybrid structure of the sensor. Figure 4b shows a SEM image of CVD grown graphene on an IDE. The CVD graphene is flat and smooth, as shown in Figure 4c. To know the quality of the as-prepared graphene, Raman spectroscopy examinations were conducted on different places. The intensity of the D band of graphene is so small that it can almost be ignored, which means that the graphene is of very good quality with little impurities and defects. The intensity ratio (I_2D_/I_G_) of as synthesized graphene is 1.61, which implies that the as prepared graphene is monolayer [44]. A SEM image of RGO0 is shown in Figure 4d. The Raman spectrum of RGO0 from the inset in Figure 4d shows that the intensity of the D band of RGO0 has increased sharply compared to graphene, which means that RGO0 contains a lot of defects.

**Figure 5:**
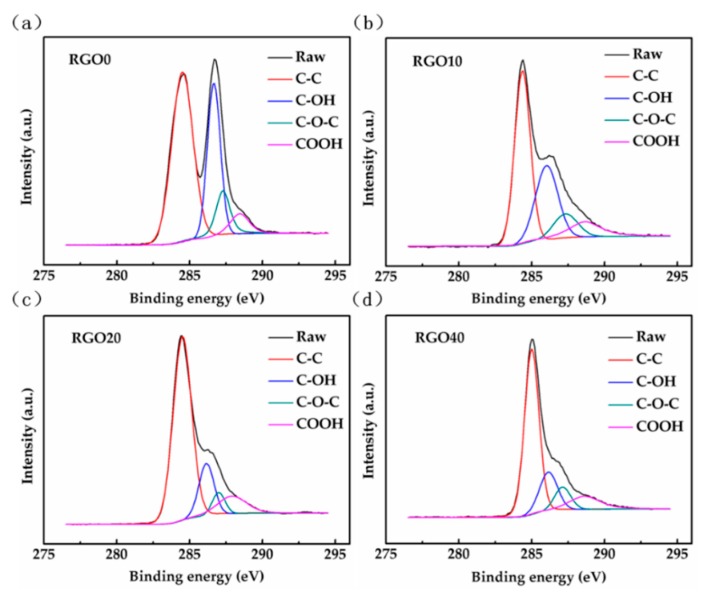
XPS spectra of Gaussian fitted peaks of (**a**) RGO0; (**b**) RGO10; (**c**) RGO20; and (**d**) RGO40, respectively.

XPS measurements have been executed on RGO0, RGO10, RGO20 and RGO40 to study the functional group changes depending on the RGO reduction time. Figure 5 shows the contents of various groups in the four RGOs with different reduction time (a, b, c and d correspond to RGO0, RGO10, RGO20 and RGO40, respectively). The relative intensity of each element as a function of binding energy is shown in Figure 5. The Gaussian fitted peaks around binding energy of 284.8, 286.5, 287.8 and 289.2 eV show the existence of carbon sp^2^ (C-C), hydroxyl (C-OH), epoxy (C-O-C) and carboxyl (COOH) functional groups in the as-synthesized RGO-Gr hybrid samples, respectively [45]. These XPS results show the surface of RGO has a mixed composition of C-C, C-OH, C-O-C and COOH. The total percentage of the three groups (C-OH, C-O-C and COOH) decreased with the RGO reduction time, and the percentage of C-OH is always the highest among the three groups, then C-O-C, and COOH is the lowest.

Meanwhile, the total percentage of the three groups (C-OH, C-O-C and COOH) in RGO10 is much more than those in the latter two RGOs (RGO20 and RGO40). The protocol and fitting results of split peak are shown in the SI. When the reduction time reaches 40 min, the reduction of RGO is almost complete. As the reduction time continues to increase, the percentages of each functional group will tend to be nearly stable.

### 3.2. Sensing Mechanism of Hybrid Structure Sensor

**Figure 6:**
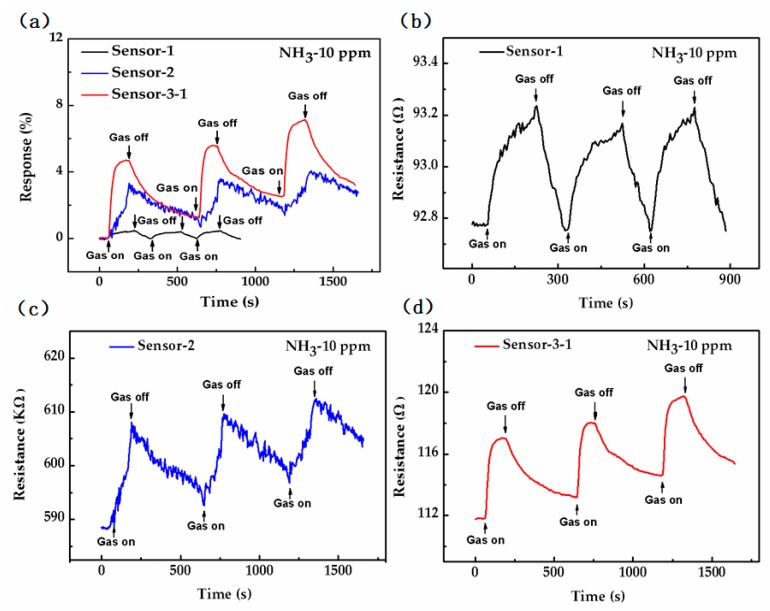
(**a**) Response of three types of sensors to 10 ppm ammonia; Resistance changes of (**b**) Sensor-1; (**c**) Sensor-2; and (**d**) Sensor-3-1 to 10 ppm ammonia gas respectively

The exposure of three sorts of sensors to ammonia gas (NH_3_) results in an increment in the electrical resistance ratio (Figure 6a). 

R_ammonia_ and R_air_ are the electrical resistance values with and without ammonia gas, respectively. Electrical resistance response is defined as a ratio expressed in percentage:

(1) $$\mathrm{Response}(\%)=\frac{R_{\mathrm{ammonia}}-R_{\mathrm{air}}}{R_{\mathrm{air}}}\times 100\%$$

The black, blue and red curves correspond to Sensor-1, Sensor-2 and Sensor-3-1, respectively. The resistances of the sensors increase dramatically when exposed to 10 ppm ammonia gas, and then drop with the ammonia gas off (i.e., air on). According to Figure 6a, Sensor-1 gives a negligible electrical resistance ratio resistance increment reading when exposed to NH_3_ (0.48%) in comparison to Sensor-2 (3.32%) and Sensor-3-1 (4.7%), as shown in the Table 2. This clearly suggests a high response of Sensor-3-1 towards NH_3_. A plausible sensing mechanism was established. CVD graphene is a p-type material with holes as major carriers under ambient conditions originating from adsorbed water or oxygen molecules [46]. The resistance of the sensor rises in the ammonia because the recombination of electrons produced by ammonia molecule adsorption and holes in graphene, which leads to a decrease of the carrier concentration in graphene. The exposure of the three sorts of sensors to continuous NH_3_ cycles has demonstrated good consistency and reversibility (Figure 6).

Although graphene has the lowest resistance (~92.78 Ω) as seen in Figure 6b, its gas sensing properties are not very good because of the lack of gas adsorption sites in its perfect lattice structure. Compared to Sensor-1 with graphene only, Sensor-2 with RGO0 only has higher resistance (~588 kΩ) as seen in Figure 6c. In contrast RGO0 has introduced a large number of adsorption sites including defects and functional groups (as shown in Figure 5a) that greatly increases the amount of gas adsorption and transferred charges in the ammonia gas as seen in Figure 6c, which causes a dramatic increase in the gas sensing response. Unfortunately, Sensor-2 with RGO0 has also introduced noise because of its low signal-to-noise ratio (SNR, calculated as shown in Equation (2) [47]) of 22 and 5.36 kΩ resistance variation (Figure 6c) which is caused by 1/f noise and Johnson noise. The 1/f noise mainly results from the changes of minority carrier number and carrier mobility caused by defects in a sensing material such as graphene [39]. Johnson noise originates from the irregular thermal motion of the carrier, and is proportional to the impedance of the sensing material [41]. The reason why Sensor-2 has obvious increase in noise is that both of its large amount of defects and high resistance (~588 kΩ) lead to a tremendous increase in 1/f noise and Johnson noise. On the contrary, Sensor-1 has a high SNR of 87 because of its fewer defects and low resistance (~92.78 Ω) from the complete lattice structure of CVD graphene.

It is well known that the gas sensitivity characteristics of sensors are mainly related to the conductivity and adsorption sites of their sensing materials [48]. The response of Sensor-3-1 (red line) is better than that of the other two because it combines the advantages of both graphene and RGO. The graphene is used as the conductive layer and electron acceptor, and electron transfer between homogenous materials is easier due to RGO0 being an electron donor and a derivative of graphene. Simultaneously, the adsorption layer of RGO0 can provide abundant adsorption sites because of its large number of functional groups and defects. Therefore, the gas sensitivity of Sensor-3-1 is the best among the three sensors as shown in Figure 6a. The noise problems of RGO0 were not observed in Sensor-3-1, because the graphene and RGO0 combined with a large region of π-π bonds, defects and functional groups in RGO0 that do not affect the graphene conductive layer. Sensor-3-1 is a hybrid structure of graphene and RGO0 and its equivalent resistance is equal to the sum of the parallel resistance values of graphene and RGO. The resistance of Sensor-3-1 (~111.7 Ω) is very close to that of Sensor-1 (~92.78 Ω), which is much less than 588 kΩ of Sensor-2. As a result, the noise introduced by Sensor-3-1 is very close to that of Sensor-1, such as the resistance variation caused by noise (0.09 Ω of Sensor-3-1 to 0.054 Ω of Sensor-1). Thus, Sensor-3-1 has very low noise because its resistance is very low close to that of CVD graphene. Sensor-3-1 has produced a SNR of 304 as shown in Figure 6d which is not only much higher than that of Sensor-2 (~22), but also higher than that of Sensor-1 (~87), because the gas sensing response of Sensor-3-1 is much higher compared to Sensor-1.

The recovery characteristics of Sensor-1 are faster than those of the other two sensors, because gas adsorption of graphene is a physisorption phenomenon with a weak binding energy, while the gas adsorption of RGO0 is a chemisorption phenomenon with strong binding energy.

The SNR could be obtained according to reference [47]. The signal power is derived from the resistance variation (∆R) during gas introduction. The power of noise is electrical noise from instrumentation before gas introduction. In this case, the electrical noise is determined by the root-mean-square (rms_noise_) value of the initial resistance (i.e., before gas introduction). The SNR could be obtained after resistance variation is divided by rms_noise_ as follows:

(2) $$\mathrm{SNR}=\frac{\Delta R}{{\mathrm{rms}}_{\mathrm{noise}}}$$

### 3.3. Effect of the RGO Reduction Time on Sensing Performance

**Figure 7:**
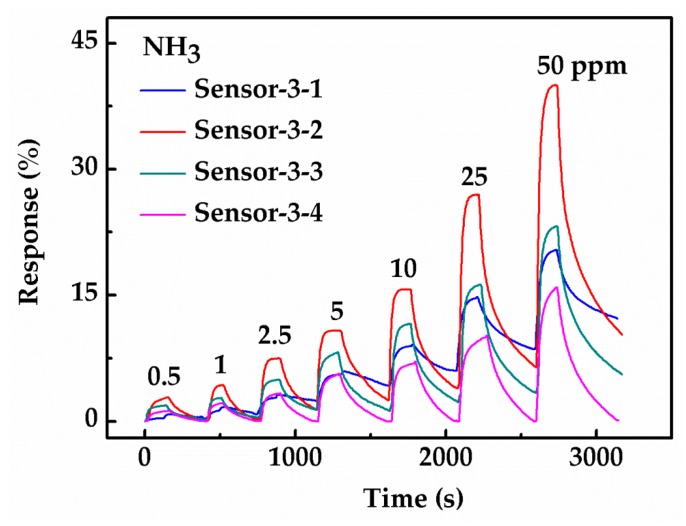
Gas sensing response of four hybrid sensors under seven different ammonia concentrations (0.5, 1, 2.5, 5, 10, 25, 50 ppm).

To study the influence of the hybrid structure on the response, comparative gas sensing experiments have been conducted on Sensor-3-1, Sensor-3-2, Sensor-3-3 and Sensor-3-4. Figure 7 shows the gas sensing response of the four sensors under seven different concentrations of ammonia (0.5, 1, 2.5, 5, 10, 25, 50 ppm). The profiles of the four sensors are similar, in which the gas sensing response gradually increases with the gas concentration. As it can be seen clearly, the response of Sensors-3-1 exhibits the least value, Sensors-3-3 with a RGO20 on graphene structure shows higher NH_3_ variation value than Sensors-3-4 with RGO40, while Sensors-3-2 with a RGO10 on graphene structure exhibits the highest response compared to the other three sensors.

**Figure 8:**
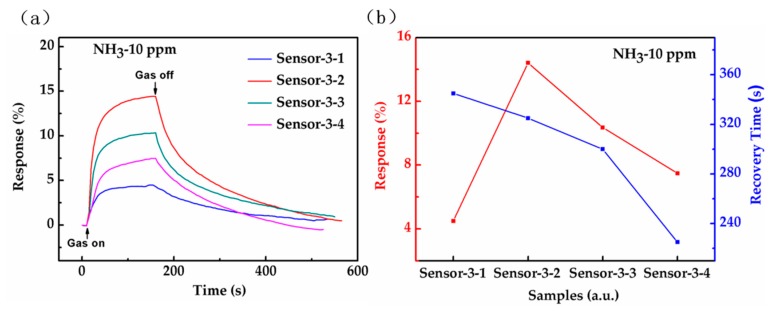
(**a**) Gas sensing response curves of four sensors to 10 ppm ammonia; (**b**) Gas sensing and recovery time of four sensors for 10 ppm ammonia.

In order to study the effects of different RGO reduction times on the hybrid structure gas sensors, the comparative response-time profiles of the four sensors are plotted in Figure 8a with a gas on (150 s)/gas off (350 s) cycle of 500 s at 10 ppm ammonia. The gas sensing response of Sensor-3-2 is the best, and the sensing response of Sensor-3-4 is more than that of Sensor-3-1, but less than that of Sensor-3-3. With the increase of RGO reduction time, the gas sensing response of the sensors increases firstly and then decreases, reaching the highest value in Sensor-3-2 (10 min of reduction time).

For one thing, RGO mainly contains three kinds of functional groups (C-OH, C-O-C and COOH), respectively. Graphene modified with each group individually constitutes a particular hybrid structure. This experiment has compared the values of current and sensitivity of three hybrid structures during ammonia molecule adsorption, as described in the SI. The conclusion can be drawn that the hybrid structure of graphene modified with C-OH outputs the highest current and sensitivity compared to the other two hybrid structures. Therefore, this experiment has extracted the ratio of C-OH and C-C groups in the Gaussian fitted peaks of the sensors Sensor-3-1, Sensor-3-2, Sensor-3-3, and Sensor-3-4, as shown in the following Table 3. Except for RGO0, the C-OH group ratio of Sensor-3-2 is the highest among the other three RGO on graphene hybrid structures, so it has the highest response to ammonia gas, as shown in Figure 8.

From Figure 5 and Table 3, Sensor-3-1 shows the highest C-OH group ratio and least response among the four sensors. RGO0 is rich in various functional groups and this results in a high resistance of ~588 kΩ. On the contrary, the amount of functional groups of the other three RGOs is less than that of RGO0 and this results in low resistances of less than 20 kΩ [46,49].

The interaction between ammonia molecules and C-OH causes a change of the resistance of the adsorbent layer, which also affects the resistance of the graphene transport layer via π-π stacking. The coupling efficiency in the hybrid structure is obviously reduced because of the high resistance of RGO0, which can counteract the advantages of a higher C-OH ratio, and attenuate the response of the sensor. Although the C-OH ratio of RGO0 is higher than that of the other three RGOs, the excessive adsorption resistance of RGO0 counteracts the transmission current of the hybrid structure. Therefore, in general, the responses of the other three RGO-Gr hybrid structures are higher than that of the RGO0-Gr hybrid structure, and Sensor-3-2 is the best among the four sensors. Due to the best gas sensing response and the use of graphene as a conductive layer without introducing additional noise, the signal-to-noise ratio of Sensor-3-2 is the highest, up to 1008.

The response and recovery times, a significant indicator for gas sensors, are defined as the time to reach 90% of the final highest value after the gas is injected and the time to reach 10% of the highest value after the gas is removed, respectively [46]. The profile shows that the response times of four sensors are very close, and are all less than 1 min. The recovery times in Figure 8b of Sensor-3-1, Sensor-3-2, Sensor-3-3, Sensor-3-4 to NH_3_ are 345 s, 325 s, 300 s and 225 s, respectively. This means that the recovery time decreases monotonically as the RGO reduction time increases.

As an almost insulator, RGO0 has a particularly poor conductivity. However, with the increase of the reduction time, the functional groups in the RGO gradually decrease, which leads to the increase in the conductivity of RGO and the recovery speed of sensors. Therefore, the recovery time decreases monotonically with RGO reduction time.

**Figure 9:**
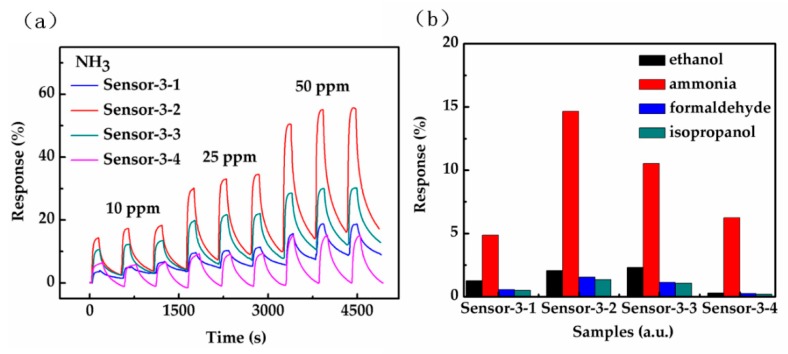
(**a**) Profile of four RGO-Gr hybrids gas sensors after three continuous cycles under three different concentrations of ammonia; (**b**) selectivity response when exposed to ethanol, formaldehyde, isopropanol and ammonia at 10 ppm.

The exposure of the four RGO-Gr hybrid gas sensors to three continuous cycles of three different concentrations of ammonia is shown in Figure 9a. As the concentration of ammonia increases, the gas sensing response increases. Meanwhile, the results perfectly match that the gas sensing response of RGO on graphene hybrid that increases first and then decreases with the increase of the RGO reduction time. All four sensors have demonstrated good consistency and reversibility under three kinds of ammonia concentration (10, 25, 50 ppm).

As for Figure 9b, RGO on graphene hybrids structure give a negligible response when exposed to organic vapours such as ethanol, formaldehyde and isopropanol compared to 10 ppm NH_3_. This clearly suggests the strong selectivity of the RGO on graphene hybrid structure towards NH_3_. The strong selectivity towards NH_3_ may be due to the strong reducing and donor behaviour of NH_3_ among the introduced vapours, as this paper shows that NH_3_ has injected a much higher amount of negative charge carriers into the RGO on graphene hybrids compared to other introduced vapours. Therefore, the negative charge carrier density of the RGO on graphene hybrids is greatly enhanced.

**Figure 10:**
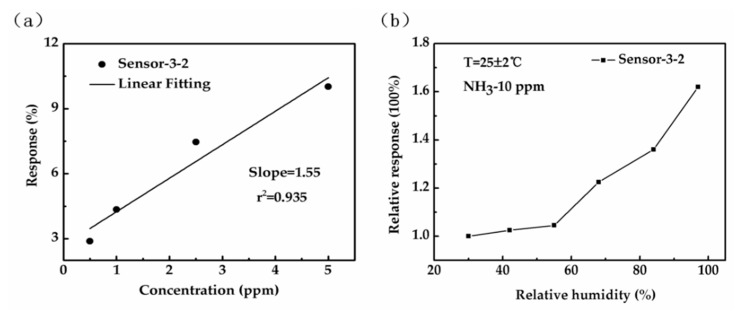
(**a**) Linear response with increase of ammonia concentration of Sensor-3-2; (**b**) relative response of Sensor-3-2 to 10 ppm ammonia under different relative humidity values.

In order to obtain the detection limit of these gas sensors to ammonia, Sensor-3-2 is selected as the reference sensor for the detection limit due to its excellent gas sensing response under all concentrations as compared to the other sensors. The linear responses with increase of NH_3_ concentration of Sensor-3-2 at seven ammonia concentrations are illustrated separately in Figure 7. Data at low concentration has been extracted in order to investigate the minimum concentration detection limit of Sensor-3-2. A straight fitting line is shown in Figure 10a. The detection limit is calculated using Equation (3) [27], and the details are given in the SI:

(3) $$\mathrm{DL}(\mathrm{ppb})=3\frac{{\mathrm{rms}}_{\mathrm{noise}}}{\mathrm{slope}}=36\;\mathrm{ppb},$$

where, ${\mathrm{rms}}_{\mathrm{noise}}$ is the root-mean-square deviation of sensor noise, and the calculated value of ${\mathrm{rms}}_{\mathrm{noise}}$ is 0.019. slope is the slope of the black line (1.55 in Figure 10a). Finally, the gas detection limit of Sensor-3-2 is calculated to be 36 ppb.

Because humidity is an important factor affecting the gas sensitivity of graphene sensors, the effect of humidity on the gas sensing properties of the graphene sensors was studied. Here, the response of Sensor-3-2 toward 10 ppm ammonia gas under different humidity conditions was tested, as shown in Figure 10b. The abscissa represents the change of relative humidity, and the ordinate represents the multiple of the gas sensing response of Sensor-3-2 under different relative humidity in response to 10 ppm ammonia. It can be seen from the profile that when the relative humidity is at a low level, the gas sensing response change of Sensor-3-2 is relatively small, while when the relative humidity reaches 68%, the gas sensing response of the sensor is enhanced significantly with the increase of relative humidity. This is due to the emergence of ammonium hydroxide (NH_4_OH) derived from ammonia molecules and water molecules on graphene, which has stronger N doping ability than ammonia, so the gas sensing response of the graphene-based sensors increases evidently [50].

The performance of our sensor was compared with several sensors recently reported by other groups. Table 4 summarizes the sensing materials, gas concentration, response and operating temperature of these studies. The first three articles mainly used polymers and their complexes with other nanomaterials as gas-sensitive materials [51,52,53]. Although it is possible to work at room temperature, the sensitivities of these sensors are not very high. SnO_2_ modified with Pt nanoparticles has a good gas-sensitive response [54], however, a high operating temperature limits its application. Interestingly, our RGO-Gr hybrids film had the best gas sensitivity among these devices at room temperature.

## 4. Conclusions

In summary, a hybrid structure gas sensor of reduced GO decorated graphene (RGO-Gr) has been designed to take advantages of both the high conductivity of graphene with its perfect lattice structure and RGO with lots of adsorption sites to realize ultra-low concentration ammonia detection. The RGO-Gr hybrid sensor not only exhibits the best gas sensing performance compared to pristine graphene or pristine RGO sensors, but also greatly improves the anti-noise ability with the SNR increasing from 22 to 1008 compared to the RGO gas sensor. In order to further improve the properties of these gas sensors, the influence of RGO reduction time on sensing properties has been investigated. The RGO10-Gr hybrid structure exhibits an excellent response of 2.88% at 0.5 ppm and good selectivity to ammonia gas in contrast to ethanol, formaldehyde and isopropanol. The gas detection limit of the RGO10-Gr hybrid structure is calculated to be 36 ppb. We believe that this work may be valuable for the future research on gas sensors.

## Figures and Tables

**Table 1:** Relationship of sensor name and sensing material.

Sensors	Sensor-1	Sensor-2	Sensor-3-1	Sensor-3-2	Sensor-3-3	Sensor-3-4
Materials	Graphene	RGO0	RGO0-Gr	RGO10-Gr	RGO20-Gr	RGO40-Gr

**Table 2:** Sensing materials and electrical parameters comparison of three sensors.

Sensor Name	Sensor-1	Sensor-2	Sensor-3-1
Materials	Graphene	RGO0	RGO0-Gr
Resistance of sensor	92.78 Ω	588 kΩ	111.7 Ω
Response	0.48%	3.32%	4.7%
SNR	87	22	304

**Table 3:** Structural and electrical parameters comparison of the RGO-Gr hybrid gas sensors.

Sensor Name	Sensor-3-1	Sensor-3-2	Sensor-3-3	Sensor-3-4
Adsorption layer/Resistance	RGO0/588 kΩ	RGO10/18.77 kΩ	RGO20/5.34 kΩ	RGO40/1.62 kΩ
Hybrid Resistance	111.7 Ω	176 Ω	167 Ω	191 Ω
C-OH/C-C	0.82	0.62	0.34	0.27
Response	4.7%	14.67%	10.49%	6.43%
SNR	304	1008	720	517

**Table 4:** Comparison of various indicators between different ammonia sensors.

Sensing Materials	Gas Concentration	Response	Operating Temperature	Reference
Graphene/PANI	20 ppm	3.65%	25 °C	[51]
Polyaniline	5 ppm	0.25%	25 °C	[52]
PANI/SnO_2_	10 ppm	5%	25 °C	[53]
Pt/SnO_2_	50 ppm	25%	115 °C	[54]
RGO/Graphene	0.5 ppm	2.88%	25 °C	This Work

## Supplementary Materials

The following are available online at http://www.mdpi.com/1424-8220/18/9/3147/s1, Figure S1: Device geometry models of graphene. (a) Pure graphene. Graphene decorated with (b) epoxy (C−O−C), (c) a hydroxyl (C−OH) and (d) carboxyl (−COOH) groups for ammonia sensing, Figure S2: (a) I-V curves and (b) the normalized I-V curves for NH3 adsorption on pure graphene, graphene decorated with an epoxy (C−O−C), a hydroxyl (C−OH), and carboxyl (−COOH), respectively.
