# Supplementary material for: A Reduced GO-Graphene Hybrid Gas Sensor for Ultra-Low Concentration Ammonia Detection
Source: Sensors (Basel). 2018 Sep 18;18(9):3147. doi: 10.3390/s18093147 (PMC6165569; doi:10.3390/s18093147)
Supplement: Supplementary file 1 [file sensors-18-03147-s001.pdf]

Supplementary Information

A Reduced GO-Graphene Hybrid Gas Sensor for Ultra-Low Concentration Ammonia Detection

Chang Wang [1,2], Shaochong Lei [1], Xin Li [1,2,*], Shixi Guo [1], Ping Cui [1], Xianqi Wei [1,3], Weihua Liu [1,2] and Hongzhong Liu [4]

[1] Department of Microelectronics, School of Electronics and Information Engineering, Xi'an Jiaotong University, Xi'an 710049, China; wangc254@163.com (C.W.); leisc@mail.xjtu.edu.cn (S.L.); guoshixi@stu.xjtu.edu.cn (S.G.); cuiping_dc@163.com (P.C.); wei.wxq@163.com (X.W.); lwhua@mail.xjtu.edu.cn (W.L.)

[2] Guangdong Shunde Xi'an Jiaotong University Academy, NO.3 Deshengdong Road, Daliang, Shunde District, Foshan 528300, China

[3] Research Institute of Xi'an Jiaotong University, No. 328 Wenming Road, Xiaoshan District, Hangzhou 311215, China

[4] State Key Laboratory for Manufacturing Systems Engineering, Xi'an Jiaotong University, Xi'an 710049, China; hzliu@mail.xjtu.edu.cn

* Correspondence: lx@mail.xjtu.edu.cn; Tel.: +86-29-8266-3343

Experiments

1. The control and calibration of ammonia concentration and relative humidity.

In this work, we used an ammonia solution instead of ammonia gas. We obtained the concentration of ammonia by the volume of ammonia solution extracted and the volume of the chamber. The specific calculation was as follows. The concentration of ammonia ($C_{ammonia}(ppm)$) was calculated by Equation (S1):

$$C_{ammonia} = V_{ammonia}/V \quad \text{(S-1)}$$

where, V was the total volume of chamber (4.67 L), $V_{ammonia}$ was the volume of ammonia gas at normal temperature and pressure, which can be obtained according to Clausius-Clapeyron equation as follows:

$$PV_{ammonia} = nRT \quad \text{(S-2)}$$

R is the gas constant. P, T represent the air pressure and temperature, respectively. n is the amount of substance, which was obtained by the following equation:

$$n = v \cdot \frac{wt.\% \cdot \rho}{M} \quad \text{(S-3)}$$

where, v was the volume of ammonia solution tested, M was the relative molecular mass of ammonia, $wt.\%$ and ρ represented the mass fraction and density of ammonia solution.

High humidity was achieved by passing air into deionized water and then into the chamber. Humidity adjustment was achieved by using a desiccant and air pump for a long period of cycle pumping and drying. When the desired humidity was reached, a certain amount of ammonia solution was injected. The desiccant absorbs the moisture of the ammonia vapor and converts it into dry ammonia gas. Although the desiccant and air pump were always pumping and drying, the humidity inside the chamber remained because of the test time for introducing ammonia gas being very short (less than 3 minutes). The relative humidity was calibrated by a humidity sensor.

2. The functional groups on the RGO different reduction times

The spectrum decomposition was performed using the XPS PEAK 41 program with Gaussian functions after subtraction of a Shirley background. We first determined the binding energy (peak position) of each functional group according to the literatures [45,47,48] and XPS spectra. We firstly fixed each peak position of these functional groups, and then optimized results to make the fitting peaks closer to the XPS spectrum as much as possible, while ensuring that the full width at half maxima (FWHM) was less than 2.7eV.

The XPS results show the surface of RGO has a mixed composition of C-C, C-OH, C-O-C and COOH. The functional groups content is directly proportional to its peak area. The peak area fitting results of each functional group in RGO are shown in the following Table S1. The total percentage of the three groups (C-OH, C-O-C and COOH) is decreasing with the reduction time of RGO, and the percentage of C-OH is always the highest among the three groups, then C-O-C, COOH is the lowest.

Table S1. Normalized peak area of functional groups in RGO.

Functional group	Normalized Peak Area (%)			
	**C-C**	**C-OH**	**C-O-C**	**COOH**
	284.8 eV	286.5 eV	287.8 eV	289.2 eV
RGO0	49.6	39.8	8.15	2.26
RGO10	51	31.92	10.2	6.17
RGO20	62.2	21.3	12.1	4.25
RGO40	69.9	19	8.1	2.88

[45]Robinson, J. T.; Perkins, F. K.; Snow, E. S.; Wei, Z. Q.; Sheehan, P. E., Reduced Graphene Oxide Molecular Sensors. *Nano Lett.* **2008**, *8,* 3137-3140.

[47]Novoselov, K. S.; Geim, A. K.; Morozov, S. V.; Jiang, D.; Zhang, Y.; Dubonos, S. V.; Grigorieva, I. V.; Firsov, A. A., Electric field effect in atomically thin carbon films. *Science* **2004**, *306*, 666-669.

[48]Bai, S. L.; Zhao, Y. B.; Sun, J. H.; Tian, Y.; Luo, R. X.; Li, D. Q.; Chen, A. F., Ultrasensitive room temperature NH_3 sensor based on a graphene-polyaniline hybrid loaded on PET thin film. *Chem. Commun.* **2015**, *51*, 7524-7527.

3. Effect of functional groups on the response of graphene to ammonia gas

In order to study the effect of functional groups (namely hydroxyl, epoxy and carboxyl, as shown in Figure S1) on the response of graphene to ammonia gas, the electron transport properties are simulated. I-V curves of graphene decorated with three kinds of functional groups are calculated, and voltage of -2 V to 2 V is applied to the device model is steps of 0.1 V.

As we can see obviously from Figure S2a, graphene decorated with a hydroxyl group has the highest I-V response and much higher than others, while pure graphene has the lowest I-V response. To make this easier to understand, the current is normalized to $|I-I_0|/I_0$, as shown in Figure S2b.

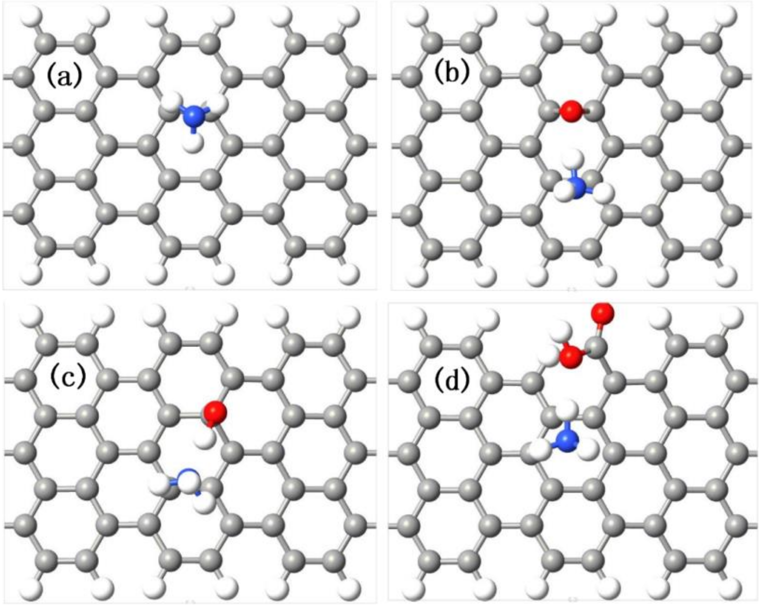

Figure S1. Device geometry models of graphene. (a) Pure graphene. Graphene decorated with (b) epoxy (C–O–C), (c) a hydroxyl (C–OH) and (d) carboxyl (–COOH) groups for ammonia sensing.

I and I_0 represent the current after and before the ammonia gas is adsorbed on the channels, respectively. Thus, the normalized current can directly represent the sensitivity in our experiments. Obviously, the sensitivity of graphene decorated with a hydroxyl group to NH_3 is found to be much higher than the sensitivity of others and it is independent of the bias voltage. In addition, graphene decorated with a carboxyl group also exhibits a higher sensitivity to ammonia gas molecule, especially at the voltage of 1.1V~1.9V, compared to pure graphene. In conclusion, the gas sensor model of graphene decorated with functional groups can enhance the sensitivity of graphene gas sensors, especially for graphene decorated with a hydroxyl group.

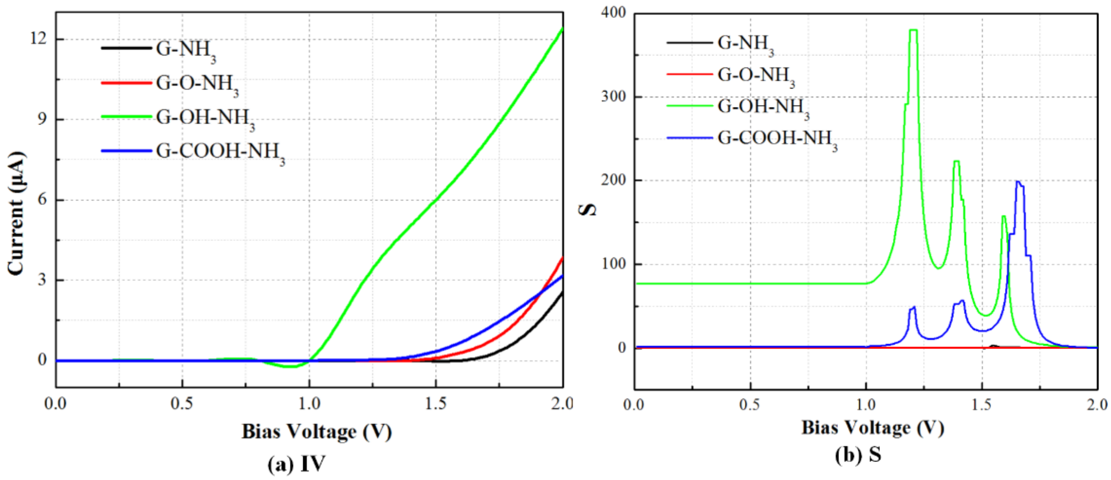

Figure S2. (a) I-V curves and (b) the normalized I-V curves for NH_3 adsorption on pure graphene, graphene decorated with an epoxy (C–O–C), a hydroxyl (C–OH), and carboxyl (–COOH), respectively.

4. Calculation of the gas detection limit

The lowest detectable concentration is limited by the present experimental setup. We can derive the detection limit from the signal processing performance of sensors as described below. The noise of sensors can be calculated using the variation in the relative conductance change in the baseline using the root-mean-square deviation [22]. We have taken 10 data points at the baseline before the ammonia exposure:

$$V_{x^2} = \sum (x_i - y)^2 \quad \text{(S-4)}$$

The rms_{noise} is calculated as follows:

$$rms_{noise} = \sqrt{V_{x^2}/N} \quad \text{(S-5)}$$

The sensor noise is 0.0186 for the sensor according to the above equation in Figure 9. When the signal-to-noise ratio equals 6, the signal is considered to be a true signal. Therefore, the detection limit can be extrapolated from the linear calibration curve when the signal equals 6 times the noise:

$$DL(ppb) = 3\frac{rms_{noise}}{slope} = 36\,ppb \quad \text{(S-6)}$$

From the above equation, the NH_3 detection limit of the sensor based on Sensor-3-2 is calculated to be 36 ppb.
